# Molecular and microscopy detection of *Pneumocystis jirovecii* in hospitalized patients during the COVID-19 pandemic

**DOI:** 10.3389/fmed.2023.1148320

**Published:** 2023-04-06

**Authors:** Roya Matouri, Shima Aboutalebian, Elahe Nasri, Somayeh Sadeghi, Soodabeh Rostami, Hamed Fakhim, Safiyeh Ghafel, Mahnaz Hosseini, Somayeh Mousavi, Faezeh Rouhi, Nader Pestechian, Hossein Mirhendi

**Affiliations:** ^1^Department of Medical Parasitology and Mycology, School of Medicine, Isfahan University of Medical Sciences, Isfahan, Iran; ^2^Mycology Reference Laboratory, Research Core Facilities Laboratory, Isfahan University of Medical Sciences, Isfahan, Iran; ^3^Infectious Diseases and Tropical Medicine Research Center, Isfahan University of Medical Sciences, Isfahan, Iran

**Keywords:** *Pneumocystis jirovecii*, COVID-19, epidemiology, nested PCR, staining

## Abstract

**Introduction:**

Early detection of *Pneumocystis jirovecii* as an opportunistic pathogen that may endanger predisposed persons, including COVID-19 patients, may help to choose the optimal management.

**Methods:**

In this study, 585, including 530 COVID-19 patients, with clinical and radiological evidence of respiratory diseases, were investigated for *P. jirovecii* screening. Clinical specimens were examined by direct microscopy and PCR, and randomly selected positive PCR products were confirmed through DNA sequence analysis.

**Results:**

Thirty-one (5.3%) samples were positive in *P. jirovecii*-specific nested-PCR, while by direct microscopic tests, *Pneumocystis* was observed in 22 (3.76%) samples. Males (61.7%) and patients over 50 years old (75.6%) were more commonly affected than others, and malaise and fatigue (84%), and wheezing (75%) were the most common symptoms, followed by fever (40.48%) and dyspnea (39.51%). Among the Pneumocystis-positive patients, three cases had coinfection with *Aspergillus fumigatus*, *A. flavus*, and *A. niger* (each *n* = 1), as documented by direct microscopy, culture, and species identification by PCR-sequencing.

**Conclusion:**

Pneumocystis pneumonia is still a diagnostic challenge; therefore, additional large-scale studies are needed to clarify the epidemiology of the disease in immunocompromised or COVID-19 patients.

## Introduction

*Pneumocystis jirovecii* (formerly *Pneumocystis carinii* f. sp. *hominis*) is a ubiquitous unicellular human-specific opportunistic fungus causing *Pneumocystis* pneumonia (PCP) in the immunocompromised hosts, such as in people living with HIV/AIDS (1, 2). In fact, after the introduction of highly active antiretroviral therapy (HAART), the population at risk for PCP shifted to non-HIV immunocompromised patients, mainly in the cases of steroid or cytotoxic therapy to treat malignancies, organ failure, solid organ transplantation, or autoimmune, inflammatory, and collagen-vascular diseases (3–5). *Pneumocystis* colonization is also increasingly identified in patients with various pulmonary conditions, contributing to more rapid declines in pulmonary function (6).

The symptoms of PCP are non-specific and include fever, non-productive cough, dyspnea, chest pain, and fatigue (7). Therefore, a diagnostic challenge occurs when a patient is colonized or infected with *Pneumocystis* or when another concurrent infection causes pulmonary dysfunction; it is sometimes unclear which disease triggers the clinical manifestations, and failure to detect one may lead to improper treatment and persistence of symptoms (1). In addition, *Pneumocystis* cannot be cultured *in vitro*, and its conventional diagnosis has relied primarily on microscopic observation of organisms in respiratory specimens with cytochemical or immunofluorescence staining (6). Several PCR assays with 10–100 times more sensitivity than microscopy have been developed for the detection of *P. jirovecii* DNA, most commonly utilizing primers for the large-subunit mitochondrial rRNA gene (mtLSU) or the multicopy major surface glycoprotein (msg) gene family (7).

In COVID-19 patients, immunomodulatory factors such as corticosteroid treatment pose a risk of secondary opportunistic infections and potentially facilitate *P. jirovecii* colonization even in patients without prior PCP predisposition (1). Therefore, the immunosuppressant used for treating SARS-CoV-2 infection might be considered a risk factor for fungal coinfection, including Pneumocystis, rather than the virus itself. In patients without previous immunosuppression, lymphopenia, high cumulative dose and long duration (for at least 3 weeks) use of steroids, immunomodulatory treatment or other risk factors yet not detected, are strongly associated with the risk of PCP development. Differentiation of COVID-19 and *Pneumocystis* infections is challenging due to overlapping their clinical signs/symptoms and radiological features, as well as limitations in laboratory diagnostics (3); both infections may have a subacute presentation with dry cough and dyspnea resulting in hypoxic pneumonia with bilateral ground-glass infiltrates, lymphopenia, and elevated lactate dehydrogenase (LDH) (8).

Some studies, primarily retrospective, have been specifically designed to investigate the coinfections by bacteria, fungi, or other viruses in COVID-19 patients (9–11). However, the coinfections and relationship between SARS-CoV-2 and *P. jirovecii* are not well-known. Considering that the COVID-19 pandemic continues to be a significant public health issue, there needs to be a heightened awareness about *P. jirovecii* among COVID−19 and immunocompromised patients since the combination of these conditions may lead to more morbidity and mortality. Therefore, this study aimed to specifically detect *P. jirovecii* in respiratory samples of COVID−19 and/or immunocompromised patients using microscopic and molecular approaches, which were obtained during the COVID-19 pandemic from the leading referral hospitals in Isfahan, Iran.

## Materials and methods

### Patients

This cross-sectional descriptive study was performed on COVID-19 patients with or without immunodeficiency, presenting with pulmonary symptoms suspected of *P. jirovecii* infection, referred to Al-Zahra and Sayyed Al-Shohada hospitals, Isfahan, Iran, from November 2020 to March 2022. Five hundred eighty-five (585) patients with respiratory signs and symptoms such as shortness of breath, cough, chest pain, and typical interstitial pulmonary infiltrates in X-ray or computed tomography (CT) scan were enrolled. Five hundred twenty-two (522) patients were hospitalized in ICU, 416 had severe respiratory signs, 106 were immunocompromised, and 530 had COVID-19 of which 51 were immunocompromised. The medical records such as the disease history and demographic, and the clinical, physiological, radiological, and microbiological data were collected and analyzed. Ethical approval of the study was obtained from the ethics committee of Isfahan University of Medical Sciences, Isfahan, Iran (IR.MUI.MED.REC.1399.1082).

### Samples

Respiratory specimens, including 273 tracheal aspirates (TA), 219 sputa, and 93 bronchoalveolar lavage (BAL) fluids, taken from 530 patients with COVID-19 [confirmed by real-time PCR (12)] and 55 patients without COVID-19, were tested. The indication for collection of BAL, TA, and sputum was determined by the pulmonologists based on respiratory symptoms, laboratory parameters, and radiological observations. Indeed, due to the conditions of the patient and to ensure the safety of the hospital staff, it was not possible to take BAL samples from many patients. Sputum samples were collected using 0.9% sodium chloride solution and sent to the laboratory. To liquefy viscose sputum samples, an equal volume of 0.5% Pancreatin was added to the sample and incubated at 37°C for 1 h. All respiratory samples were centrifuged at 3000 rpm for 15 min, and the sediments were subjected to molecular/microscopic investigations.

### Molecular detection

The total DNA was extracted from 200-500 microliters sample sediments either with Add Prep Genomic DNA Extraction Kit (AddBio, Korea) or manually as described previously (13). The DNA was eluted in 30 μl distilled water and stored at –20°C until used in PCR amplification.

Specific nested-PCR was used to detect *P. jirovecii* with already described primers (14) targeting the mtLSU region. For the first stage of amplification, the external primers pAZ102-H (5′-GATGGCTGTTTCCAAGCCCA-3′) and pAZ102-E (5′-GTGTACGTTGCAAAGTACTC-3′) resulting in a 346 base pair (bp) fragment, and for the second stage, the internal primers pAZ102-X (5′-GTGAAATACAAATCGGACTAGG-3′) and pAZ102-Y (5′-TCACTTAATATTAATTGGGGAGC-3′) yielding a final fragment of 267 bp, were used. The first round of PCR contained 7.5 μl of 2× master mix (Taq DNA Polymerase 2 × Master Mix Red, Ampliqon, Denmark), 0.5 μM primers, 4 μl DNA template in a total volume of 15 μl. The reaction was run as: 95°C 5 min, 35 cycles of 95°C 30 s, 58°C 45 s and 72°C 45 s, and a cycle of 72°C 5 min. The second PCR contained 7.5 μl master mix, 0.33 μM primers, and 2 μl of the first PCR product diluted 1/50 with distilled water to a final volume of 15 μl, with the conditions: 95°C 5 min, 30 cycles 94°C 30 s, 58°C 45 s, and 72°C 30 s, and a cycle of 72°C 2 min. Five microliters of each nested-PCR product was electrophoresed, stained, and visualized under ultraviolet light. A plasmid containing the PCR product was constructed using the PCR 2.1-TOPO TA cloning kit (BIO BASIC, United Kingdom) based on the manufacturer’s instructions, and dilutions of the extracted DNAs were prepared as a positive control. Water was considered as the negative control in DNA extraction and both two PCR reactions.

Randomly selected positive PCR products were purified and subjected to sequencing (Core Facilities Laboratory, Isfahan, Iran) with the primer pAZ102-X, using BigDye Terminators (Applied Biosystems), and the sequences were subjected to BLAST analysis.^1^

### Direct microscopy

Smears were prepared from the centrifuged sediment of all PCR positive and 190 randomly selected PCR negative samples, subjected to staining with Giemsa (15), Periodic Acid-Schiff (PAS), and Toluidine Blue O (TBO) (16), and microscopically observed by an experienced laboratory technician.

### Statistical analysis

Descriptive data were analyzed as frequencies and percentages, the associations were evaluated using the chi-square test in SPSS version 22, and the results were discussed on a 5% level of significance (*P* < 0.05).

## Results

Among 585 patients ranging from 1 to 90-year-old included in this study for the examination of *P. jirovecii*, the patients > 50 years of age (75.6%) and the males (*n* = 361, 61.7%) were more frequently infected. 530/585 (90.6%) patients were admitted due to COVID-19, and 106/585 patients (18.12%) were immunocompromised because of tumors (*n* = 60, 10.3%) and hematopoietic diseases (*n* = 46, 7.8%) of which 51 were with COVID-19.

**FIGURE 1:**
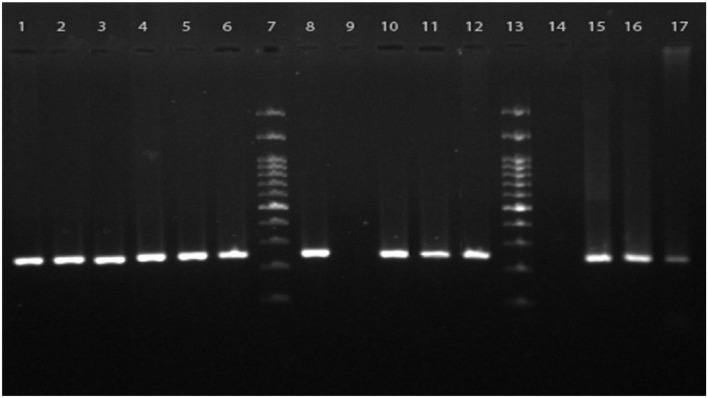
Agarose gel electrophoresis of the PCR products from clinical samples. The nested PCR was performed using the primers targeted mt-LSU ribosomal RNA for specific amplification of *Pneumocystis jirovecii* presented in respiratory specimens. Lanes 1–6, 10–12, and 15–17 are cumulative positive samples having specific 267 bp bands representative of *P. jirovecii*, lanes 8 and 9 are positive and negative controls, respectively, lane 14 is a representative of the negative clinical samples, and lanes 7 and 13 are 100 bp DNA molecular size markers.

**FIGURE 2:**
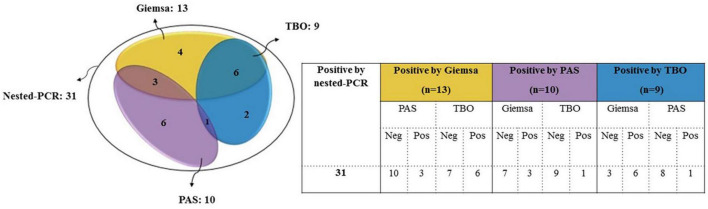
Comparison of nested PCR with microscopy results for detecting *P. jirovecii* in clinical respiratory specimens. Only those samples that had positive PCR results were included in this comparison. There was no sample with positive microscopy and negative PCR.

**FIGURE 3:**
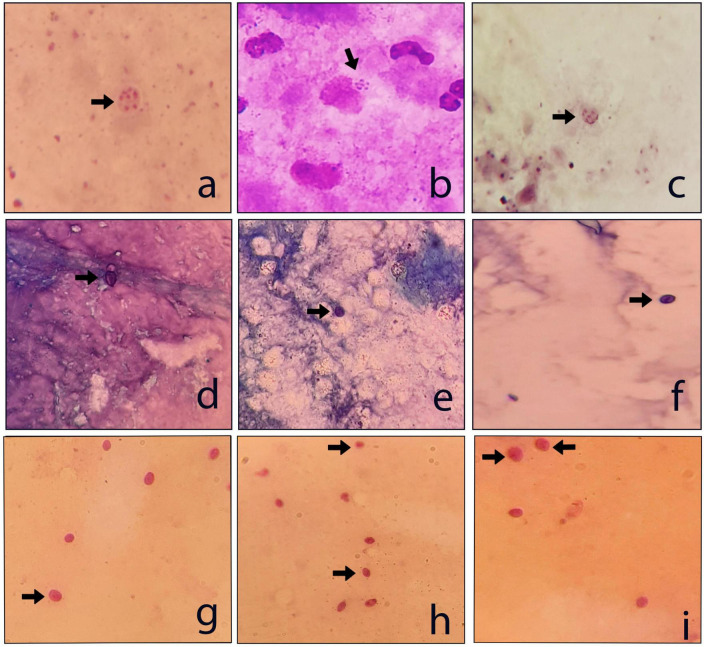
Examples of the *P. jirovecii* positive respiratory samples. **(a–c)** Giemsa stain; **(d–f)** TBO stain; and **(g–i)** PAS stain (×1000).

A total of 31 (5.3%) samples were positive in *P. jirovecii*-specific nested-PCR (Figure 1), while by direct microscopy, *P. jirovecii* was observed in 22 (3.76%) samples (Figures 2, 3). Therefore, considering the result of PCR, a 5.3% prevalence of *P. jirovecii* infections/colonization was rated. The BLAST sequence analysis of 12 randomly selected positive PCR products confirmed that all are *P. jirovecii*. No significant association between the prevalence of *Pneumocystis*-positive patients and the patient’s gender and age were found. The frequency of *Pneumocystis* in immunocompromised patients without COVID-19, immunocompromised patients with COVID-19, and other COVID-19 patients was 14.55% (*n* = 8/55), 3.92% (*n* = 2/51), and 4.38% (*n* = 21/479), respectively.

Malignant patients showed higher rates of *Pneumocystis* infection/colonization (9.43%) than other patients with various lung diseases (4.38%). Likewise, patients with acute myeloid leukemia and chronic lymphocytic leukemia had a higher infection rate (9.43% vs. 4.38%) than the other malignant patients. Interestingly, among the Pneumocystis-positive patients, three cases had coinfection with *Aspergillus fumigatus*, *A. flavus*, and *A. niger* (each *n* = 1), as documented by direct microscopy (KOH preparation), culture, and species identification by PCR-sequencing (17).

All 31 patients with positive PCR results showed at least some clinical symptoms of *P. jirovecii* pneumonia. Malaise and fatigue (84%) (although they are not specific symptoms), wheezing (75%), fever (40.48%), dyspnea (39.51%), cough (36.1%), and sepsis (15.12%) were the most common clinical manifestations. The most common host predisposing factors were diabetes mellitus (9.75%), hypertension (9.26%), chronic kidney (9.26%), and pleural effusion (7.21%). All positive patients were treated with Co-Trimoxazole, and no remarkable side effect was reported.

The relationship between *Pneumocystis* PCR-positive and the clinical manifestations was analyzed in which malaise and fatigue (*P* = 0.037), wheezing (*P* = 0.007), fever (*P* = 0.000), and dyspnea (*P* = 0.000) were significantly associated with the pneumocystosis.

**FIGURE 4:**
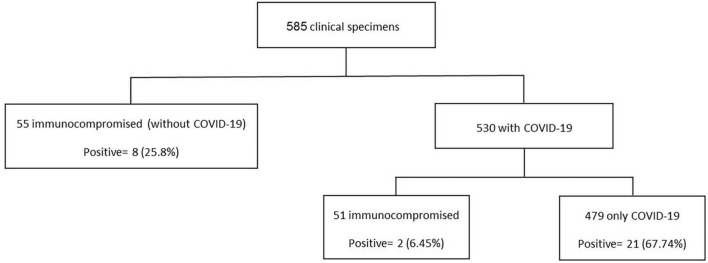
Flowchart of patients investigated in this study.

As shown in Table 1, from 31/585 patients with *P. jirovecii*, 21 (67.74%) had COVID-19, eight (25.8%) had cancer, and two (6.45%) had both COVID-19 and cancer (Figure 4). The average age in the positive cases was 54.21 years (range 1.5–82 years), and the males (*n* = 23, 79.19%) were more frequently infected than the females. Nineteen positive patients died, and three were cured; for the remaining nine patients, the information was not available. Of the 19 who died, eleven had COVID-19, seven had cancer, and one case had cancer and COVID-19 together. According to the type of tested sample, most positive samples were observed over the TA (*n* = 17/273, 6.23%), followed by sputa (*n* = 12/219, 5.48%) and BAL (*n* = 2/93, 2.15%).

**TABLE 1:** Features of 31 patients positive in *Pneumocystis jirovecii*-specific nested-PCR.

Patient	Sex	Age	Type of tested sample	Underlying disease or condition	Microscopy result (Giemsa or TBO or PAS)	Outcome
1	F	65	Trachea	Malignant multiple myeloma	Neg	Died
2	M	55	Trachea	Malignant acute myeloblastic leukemia	Neg	Died
3	M	71	Trachea	Malignant neoplasm of kidney, except renal pelvis + COVID-19	Pos	Died
4	M	71	Trachea	Malignant acute myeloblastic leukemia	Pos	Cured
5	M	67	Trachea	Malignant large B-cell lymphoma + COVID-19	Pos	Cured
6	F	47	Trachea	Malignant neoplasm of breast	Pos	Died
7	F	80	Trachea	Malignant neoplasm of the thyroid gland	Pos	Died
8	M	78	Sputum	COVID-19	Pos	Died
9	F	8	Sputum	COVID-19	Pos	Cured
10	M	26	Sputum	COVID-19	Pos	Died
11	M	6	Sputum	COVID-19	Pos	Died
12	M	49	Sputum	COVID-19	Pos	Died
13	F	68	Sputum	COVID-19	Pos	Died
14	M	39	Trachea	Malignant neoplasm of the prostate	Neg	Died
15	M	47	Trachea	COVID-19	Neg	NA
16	M	32	Sputum	COVID-19	Pos	NA
17	M	50	Trachea	COVID-19	Neg	Died
18	M	49	Sputum	COVID-19	Pos	NA
19	M	68	Sputum	COVID-19	Pos	NA
20	M	55	Trachea	COVID-19	Pos	Died
21	M	72	Trachea	COVID-19	Pos	NA
22	M	68	Trachea	Malignant lymphoma	Pos	Died
23	M	59	Sputum	COVID-19	Pos	NA
24	F	76	Trachea	COVID-19	Pos	Died
25	M	78	Trachea	COVID-19	Pos	Died
26	M	67	Trachea	Malignant multiple myeloma	Neg	Died
27	F	1.5	BAL	COVID-19	Neg	NA
28	M	41	BAL	COVID-19	Neg	NA
29	M	40	Tracheal	COVID-19	Pos	Died
30	M	82	Sputum	COVID-19	Pos	Died
31	M	65	Sputum	COVID-19	Neg	NA

M, male; F, female; Pos, positive; Neg, negative; NA, not available.

## Discussion

Without timely diagnosis and treatment, *Pneumocystis* pneumonia can be a life-threatening infection (18). The most challenging issue in research on *Pneumocystis* is the lack of an *in vitro* culture method for isolation of the organism; thus, its diagnosis is presumptively based on the clinical features and the chest X-ray in the predisposed patient with a CD4 < 200 cells/μL. Nevertheless, various microscopy methods are proposed for the observation of *P. jirovecii* in stained smears, including Giemsa (15), Gomori methenamine silver (GMS) (19), toluidine blue O (16), Calcofluor white (20), or immunofluorescent staining with monoclonal antibodies (21). Recently, conventional PCR (22), nested-PCR (14), probe-based (20) and SYBR-based real-time PCR (23), 1-3-β-D-glucan assay (21), and newly next-generation sequencing (NGS) (19) have been used for detection of Pneumocystis; each method has its advantages and limitations.

The present work is one of the broadest molecular epidemiological studies to describe *Pneumocystis* colonization/infection in COVID-19 patients with or without immunodeficiency. As nested PCR is one of the most sensitive molecular methods, and more clinical cases can be diagnosed, we utilized nested PCR to detect *P. jirovecii*, in which, among 585 patients, 31 (5.3%) were positive (Table 1). However, *P. jirovecii* colonization is also probable by this method that should be considered in evaluating the cases. At least, a negative nested PCR may confidently exclude PCP.

*Pneumocystis* may persist in humans, and the epidemiological implications of this phenomenon are not yet well known. Colonization with *P. jirovecii* has been described in immunocompetent patients, especially those with chronic respiratory disorders (19). Therefore, the pathogenic impact of colonization on immunocompetent patients may not be significant as long as the patient’s immune status has not deteriorated. Nevertheless, although colonization of *P. jirovecii* does not indicate that the person has PCP, it may affect the pathophysiology of respiratory diseases suggesting that the person is at risk for *P. jirovecii* disease (24). In addition, colonized patients may represent a reservoir for transmitting *P. jirovecii* to humans, at least in indoor environments such as hospitals and perhaps the general population (25). Also, it may lead to the selection of mutations that have been associated with drug resistance in individuals receiving long-term anti-*Pneumocystis* prophylaxis; and may stimulate a host inflammatory response that leads to lung disease such as chronic obstructive pulmonary disease (COPD), and in children may be associated with conditions such as bronchiolitis and sudden infant death syndrome (26). Studies have reported 20–40% of *P. jirovecii* colonization in different patient groups (27); more recent studies have revealed a wider range of colonization/infection rates varying from 2.6 to 55% (25). However, it is difficult to compare these results because the demographic and medical data distribution, especially age and underlying diseases, are scattered.

Recently, with the rise of the COVID-19 pandemic, PCP has become more critical. It is because of the immunological imbalances, use of corticosteroids followed by respiratory impairment, and viral-induced or iatrogenic lymphopenia (28), all are the risk factors for *P. jirovecii* infection. It is essential to consider PCP alongside COVID-19 as a part of the initial differential diagnosis when screening high-risk populations presenting with a chest infection (29), however, the clinical overlap with COVID-19 impacts the accuracy of the clinical case definition for PCP (15). In our patients, the most common clinical presentations were malaise and fatigue, wheezing, fever, dyspnea, cough, and hypoxemia, which were similar to the diagnostic criteria of PCP but are not specific symptoms (30). A remarkable symptom in our study was malaise and fatigue, whereas dry cough was more prevalent in other studies (31, 32). As differentiating PCP from COVID-19 is complex, applying laboratory tests is essential.

During the spread of COVID-19, some coinfection with COVID-19 and *P. jirovecii* was reported. Kelly et al. (33) reported the coinfection rate of COVID-19 and pneumocystosis as high as 22% (15 out of 69), while in other studies, it was 1.4–9.3% (34, 35). The rate of co-existence of *Pneumocystis* with COVID-19 in our patients was 4.34% (23 out of 530). Most patients infected by *P. jirovecii* were reported in the age group of >50 years (34); our patients ranged from 1 to 90 years, and 75.6% were over 50. It is reported that the most critical risk factors for developing *Pneumocystis* pneumonia are the use of glucocorticoids, age, defective immunity, and comorbid pulmonary conditions (36). All positive patients in this study had the risk factors for developing PCP. Also, in this study, *P. jirovecii* was more commonly diagnosed in males than females (79.19% vs. 20.81%), which is in agreement with other reports (37). The mortality rate was 86.36% in our patient, which was higher than several other studies performed in other developing countries (32). Indeed, 19 of 31 patients died, and the information for 9 patients was not available. Of the 19 who died, 11 (57.89%) patients had COVID-19, seven (36.84%) were immunocompromised, and one (5.26%) was an immunocompromised COVID-19 patient. Whether this high mortality is due to COVID-19, Pneumocystis, or both remains unknown. Chi-square analysis showed a statistically significant relationship between mortality and positive results of the PCR (*P* = 0.000). We found three cases of coinfection with *Aspergillus* and *Pneumocystis* in COVID-19 patients. As far as we know, these are the first reported cases.

In this study, the number of positive samples in PCR (*n* = 31) is more than the sum of all staining methods (Table 1), which confirms the previous reports indicating the superiority of PCR for diagnosing PCP. For example, a recently published prospective blind study on BAL specimens from HIV-infected patients showed that PCR is a sensitive and reproducible test for detecting *P. jirovecii* (38). Among the stains used in this study, Giemsa was more sensitive (13 out of 22) than PAS (10/22) and TBO (9/22) (Figures 2, 3); for this reason, most guidelines prefer nested PCR rather than smear staining for selective diagnosis (26).

This study has limitations, primarily due to the critical conditions and the high workload in COVID-19 epidemics. Short−term follow−up, lack of enough clinical pictures and laboratory data such as LDH despite being an essential prognostic tool and an assessment of response to PCP therapy, and not performing quantitative PCR were among the limitations of our work.

## Conclusion

As one of the extensive epidemiological studies, *Pneumocystis* colonization/infection was PCR-detected in 31 out of 585 patients with or without immunodeficiency. While early detection of *Pneumocystis* infection/colonization and improving clinician awareness about the infection is essential to better outcomes for the patient, it remains a diagnostic challenge. Performance of high-quality and large-scale studies are needed to clarify the epidemiology of *P. jirovecii* in immunocompromised and COVID-19 patients, as during the COVID-19 pandemic, other respiratory infections including pneumocystosis, may be overlooked due to the similar symptom.

## Data availability statement

The original contributions presented in this study are included in the article/supplementary material, further inquiries can be directed to the corresponding author.

## Ethics statement

The studies involving human participants were reviewed and approved by the Ethics Committee of Isfahan University of Medical Sciences, Isfahan, Iran (IR.MUI.MED.REC.1399.1082). Written informed consent to participate in this study was provided by the participants’ legal guardian/next of kin.

## Author contributions

RM, SA, SG, MH, and SM performed all the experiments. RM, SA, and NP participated in the data collection, analysis, and interpretation. SA, RM, and FR drafted the manuscript. EN, SR, SS, and HF participated in collecting the clinical specimens. HM designed the study, supervised all parts of the study, and the critical review of the manuscript. All authors approved the final version of the manuscript.

## Footnotes

1
https://blast.ncbi.nlm.nih.gov/Blast.cgi
